# Semi-analytical Galerkin modeling of glucose transport and reaction in glucose oxidase-immobilized SBA-15 mesoporous silica

**DOI:** 10.3389/fchem.2026.1848563

**Published:** 2026-06-09

**Authors:** Jayalal J. Nair, T. Praveen

**Affiliations:** Department of Mathematics, School of Advanced Sciences, Vellore Institute of Technology, Vellore, Tamilnadu, India

**Keywords:** enzyme immobilization, glucose biosensors, glucose oxidase, reaction-diffusion modeling, SBA-15, semi-analytical Galerkin modal method

## Abstract

The immobilization of glucose oxidase (GOx) in ordered mesoporous silica can provide a stable and efficient platform for glucose sensing and biocatalytic applications. This work examines a reaction-diffusion model governing glucose transport and enzymatic conversion inside GOx-loaded SBA-15 structures, with attention to both rod-shaped and cuboid particle geometries. Three coupled phenomena are incorporated into the model: intraparticle Fickian diffusion, Michaelis-Menten kinetics modified by competitive inhibition, and the dynamic exchange between glucose within the pore and in the surrounding bulk solution. The governing system of nonlinear PDE and ODE is solved in two complementary ways: the Method of Lines (MOL) and the semi-analytical Galerkin modal method. There is a high level of concordance between the semi-analytical and numerical solutions in a wide range of combinations in enzyme loadings and pore geometries shown in simulation findings. Absolute and relative errors remain minimal, particularly under diffusion-limited conditions at high enzyme loadings. Model predictions are further validated against experimental bulk depletion data reported in the literature, with mean absolute percentage error below 1% across the examined time window. A sensitivity analysis was performed to identify that the competitive inhibition constant has a more significant impact on glucose conversion within the parameters examined compared to the effective diffusivity. The semi-analytical galerkin formulation reduces the spatial problem to a small set of modal coefficients rather than the tens-to-hundreds of nodes required by Method of Lines, yielding a compact representation well-suited for parametric and sensitivity studies without loss of accuracy in the regimes examined. The results demonstrate that the semi-analytical Galerkin modal method offers a reliable alternative to conventional numerical approaches, yielding practical design guidance for mesoporous glucose biosensors on particle geometry, enzyme loading, and inhibition mitigation.

## 1 Introduction

Glucose monitoring drives a high demand for biosensors. Glucose biosensors dominate the biosensor market with a share of approximately 85%, with a market valuation of approximately $5 billion (Newman and Turner, 2005). GOx is the enzyme used in the majority of glucose biosensors due to its high specificity and stability (Wilson and Turner, 1992). Glucose oxidase, or (β-D-glucose:oxygen 1-oxidoreductase), catalyses the oxidation of glucose to gluconolactone and H_2_O_2_ (Wilson and Turner, 1992). Furthermore, GOx has been utilized in biosensors due to its high turnover rate and robustness (Wilson and Turner, 1992). GOx has been used in biosensors since the 1980s (Newman and Turner, 2005).

The utilization of GOx in practical sensors demands immobilization on a solid support. The advantages of immobilizing enzymes are that it enables reuse, prolongs enzyme lifetime, and supports the integration into electrode platforms (Zhao et al., 2006). Compared to the free enzyme in solution, in order to enhance GOx operational stability, porous solids and polymer membranes have been utilized to entrap or attach it (Zhao et al., 2006). Researchers have investigated various supports and methods to overcome the challenges of immobilizing the GOx without losing its activity. Where supports mainly include polymeric films, nanoparticles, and porous oxides, on the other hand, methods incorporate physical adsorption, covalent binding, and cross-linking for the immobilization of GOx (Tran and Balkus, 2011; Zhao et al., 2006).

In the mid-1990s, scientists at Mobil oil discovered a family of mesoporous silicas, known as the M41S family (e.g., MCM-41) that contained pores of a uniform size between 2 and 3 nm in diameter with high degree of surface area (Taguchi and Schüth, 2005). Not long after the discovery of MCM-41, another silica with larger pores of approximately 6–10 nm in diameter, known as SBI-15 was also discovered. Ordered mesoporous silicas, such as MCM-41 and SBA-15 have surface area of around 600–1,300 m^2^/g with pore diameters that can be tuned to between 2 and 30 nm in diameter (Taguchi and Schüth, 2005), both properties of which make these materials ideal candidates for the incorporation of biomolecules. These materials have been recognised as possessing the potential to act as excellent supports for enzymes (Taguchi and Schüth, 2005). The well-defined pore structures of SBA-15 allow for the confinement of enzyme molecules within the matrix and can accommodate high enzyme loadings due to its large internal surface area. Moreover, enzyme immobilization within the porous matrix greatly facilitates enzyme separation from the reaction mixture, allows for its reuse, and minimizes enzyme leaching - all with only modest losses of enzymatic activity (Zhao et al., 2006). Furthermore, Zhao et al. state that any decrease in enzymatic activity resulting from enzyme immobilization can be outweighed by the advantages of easy enzyme recovery, enzyme reuse, and continuous enzyme operation in reactors (Zhao et al., 2006). These properties of porous silicas enable the benefits of both homogeneous and heterogeneous catalysis to be realized simultaneously, encouraging numerous research studies on the development and use of GOx-silica systems (Tran and Balkus, 2011).

To improve the performance of glucose biosensors, the mesoporous supports created the bienzymatic system within the pores. In 2008 Dai et al. achieved a “bienzyme channeling” sensor co-immobilized with GOx and horseradish peroxidase (HRP) in SBA-15; here, GOx generates H_2_O_2_ from glucose, and HRP immediately reduces H_2_O_2_ at the electrode, and this internalized cascade led to a fast, sensitive amperometric response and a wide linear glucose range (Dai et al., 2008). Wang et al. in 2009 immobilized the glucose oxidase onto the modified electrode with Nafion and SBA-15 and showed that mesoporous materials also promote direct electron transfer from GOx (Wang et al., 2009). From this study they discovered the modified electrode enhances the electron transfer and enzyme activity and also gives better glucose detection (Wang et al., 2009). The pore structure of enzyme-silica systems is another important determinant of their performance. Studies in the 2010s investigated how the properties of the mesoporous silicas could be tuned. For instance, Chen et al. synthesized platelet SBA-15 silica nanoparticles with short channels within the silica structure and enlarged pores to enable the adsorption of enzymes onto the particles (Chen et al., 2011). These platelets allowed for faster uptake of the enzymes by the particles and for each unit area of the particles to load more of the enzymes. Ding et al. prepared rod-shaped SBA-15 particles that had lengths of the mesoporous channels that could be tuned (Ding et al., 2013). The shorter lengths of the channels and the larger diameters of the pores created by these tuning strategies enabled the preservation of the catalytic activity of the immobilized enzymes.

Comprehensive reviews of the topic, such as that by Hartmann and Kostrov (2013), have discussed the benefits and challenges of enzyme immobilization on porous silicas. Specifically, Hartmann and Kostrov (2013) state that the chosen size of the pores of the porous silica can boost the activity of the immobilized enzyme. For instance, Khan et al. found that using an amino-functionalized form of mesoporous silica with pores of around 11 nm increased the response of a glucose oxidase (GOx) biosensor compared to using microporous silica (Khan et al., 2014). Moreover, the mesopores allowed for the enzymes to be contained within the pores, thus allowing for a range of glucose concentrations between 3.1 and 13 mM to be detected (Khan et al., 2014). Another study that attempted to provide further insight into this process is that by Zadeh et al. , who created a fluorescence assay that could be used to monitor the uptake of the enzyme into the mesoporous silica in real time (Zadeh et al., 2015).

Mathematical modeling was employed to gain insights into the interplay between the various processes occurring within the porous supports. Grénman et al. investigated the importance of modeling the kinetics of the reaction that occurred between the solid and liquid phases of the enzyme system, including the resistance to mass transfer within the solid phases of the system (Grénman et al., 2011). Majumdar et al. performed experiments and utilized a mathematical model to investigate the diffusion of glucose and the reaction of that glucose with GOx in SBA-15 particles (Majumdar et al., 2016). The authors found that increasing the loading of the enzyme into the porous support structure led to limitations in the diffusion of the enzyme and reduced activity of each enzyme within the system (Majumdar et al., 2016). As another example of biosensors that utilized porous supports, Khan et al. created a biosensor based on carbon paste with GOx immobilized onto powder composed of mesoporous silica (Khan et al., 2016). Balistreri et al. reported the covalent immobilization of GOx onto mesocellular foams (MCFs), which increased the stability of the enzyme in the presence of heat and solvents (Balistreri et al., 2016). Other efforts have employed the co-immobilization of both GOx and H_2_O_2_ within the same silica matrix to catalyse the oxidation of glucose (Pitzalis et al., 2017). More recently, Shin et al. have investigated the effect of confining space on glucose oxidase within different silica matrices, such as SBA-15 and MSU-F silicas (Shin et al., 2020). Finally, another report that used mesoporous silica utilized the immobilization of Orange Carotenoid Protein onto the silica for use in optoelectronic applications (Leccese et al., 2022). In 2023, Fornerod et al. stated, enhanced GOx loading and sensing performance in surface-modified aluminosilicate SBA-15 analogues (Fornerod et al., 2023).

Addressing coupled nonlinear reaction–diffusion systems present significant challenges, particularly when a partial differential equation governing glucose transport is associated with an ordinary differential equation that characterizes adsorption–desorption kinetics. The Method of Lines (MOL) transforms the spatially discretized PDE–ODE system into a collection of ordinary differential equations, which can be addressed with conventional time integrators (Schiesser and Griffiths, 2009). MOL has been extensively utilized in chemical and biochemical transport issues and is backed by various MATLAB-based toolboxes (Schiesser and Griffiths, 2009; Wouwer et al., 2004). Semi-analytical Galerkin modal methods offer a semi-analytical approach by expressing the spatial dependence of the PDE through basis functions and determining the modal coefficients with high precision (Boyd, 2001; Trefethen, 2000). These methods demonstrate effectiveness for smooth concentration fields and enable systematic error and convergence analysis (Trefethen, 2000).

Although MOL is well-established and reliable, it treats the spatial operator purely as a discrete algebraic object, which obscures the modal structure of the underlying diffusion problem and scales in cost with the number of spatial nodes. The Galerkin modal approach retains the spectral decomposition of the diffusion operator explicitly, so each retained mode carries interpretable physical meaning in terms of spatial frequency content. In stiff regimes where the concentration field is smooth, only a handful of modes are required to reproduce the MOL solution to within sub-percent error, and the resulting low-dimensional ODE system integrates substantially faster. These properties, rather than raw accuracy, are the basis for preferring the modal formulation in parametric and sensitivity studies of immobilised-enzyme systems.

Recent modeling and characterization efforts continue to advance the understanding of reaction–transport coupling in porous-support systems relevant to biocatalysis and biosensing. Arefi et al. (2025) integrated dimensionless analysis with a reaction-diffusion model in spherical coordinates to predict gel-front progression in alginate microgels synthesized under continuous flow, providing a methodological framework directly paralleling the non-dimensional reaction-diffusion treatment adopted in the present work. Chen et al. (2026) reported a dendritic mesoporous silica nanoreactor co-immobilizing GOx with Fe_3_O_4_ and Ir nanoparticles for one-step colorimetric glucose detection, demonstrating how spatial confinement within a mesoporous silica framework enhances oxidase-driven cascade catalysis and substrate channeling - a system closely related to the GOx-loaded SBA-15 particles examined here. These studies emphasize the value of coupled reaction-diffusion modelling and of mesoporous silica as an enzyme-immobilization host. The present work contributes to this body of literature by adopting a semi-analytical Galerkin modal formulation for the coupled reaction-diffusion problem within GOx-loaded SBA-15 particles, providing a computationally efficient framework for parametric and sensitivity studies in the SBA-15 biosensor regime.

This study adopts the reaction–diffusion model proposed by Majumdar et al. (2016) and solves it using two complementary techniques: a numerical solution based on the Method of Lines (MOL) and a semi-analytical Galerkin modal method (Alfifi, 2021). Both methods are used on rod-shaped and cuboid SBA-15 geometries to find out how glucose concentration changes over time in the porous enzyme-loaded domain. The profiles derived from the MOL and the semi-analytical method are compared to evaluate the concordance between the two formulations, with the close alignment of the curves validating the semi-analytical solution. An error analysis, encompassing both absolute and relative errors, is carried out to evaluate the accuracy and convergence characteristics of the methods. A normalized sensitivity analysis is also performed to determine those parameters that have the most influence upon the transport and reaction of glucose within the mesoporous structure. This study establishes a comparative framework between numerical and semi-analytical methodologies for reaction-diffusion modelling in immobilized enzyme systems, offering insights for the design and optimization of mesoporous biocatalysts and biosensing materials.

The contribution of the present work can be summarized along three complementary dimensions. From a methodological standpoint, this work formulates a semi-analytical Galerkin modal solution for the coupled nonlinear reaction-diffusion system that governs glucose transport, Michaelis-Menten kinetics with competitive product inhibition, and bulk-pore exchange in GOx-loaded SBA-15. The modal projection retains the spectral structure of the diffusion operator and integrates the nonlinear reaction term directly, yielding a formulation that is suitable for stiff, smooth concentration fields without requiring fully numerical spatial discretization. From a computational standpoint, the modal expansion truncated at twenty sine modes reduces the spatial degrees of freedom from the tens to hundreds of nodes typical of MOL discretizations to a small set of modal coefficients, producing a reduced-order ODE system that integrates substantially faster than direct discretization at comparable accuracy in the regimes examined. From an application standpoint, the framework is applied to two SBA-15 morphologies most commonly reported in the biosensing literature, namely, rod and cuboid particles, under realistic enzyme loading and bulk concentration conditions, and the resulting parametric and sensitivity behavior is translated into concrete design guidance covering particle geometry selection, enzyme loading strategy, and the relative priority of inhibition mitigation versus diffusion-path optimization. These three contributions are interdependent: the methodological formulation enables the computational reduction, and the computational efficiency enables the application-level parametric study.

## 2 Formulation of the problem

**FIGURE 1:**
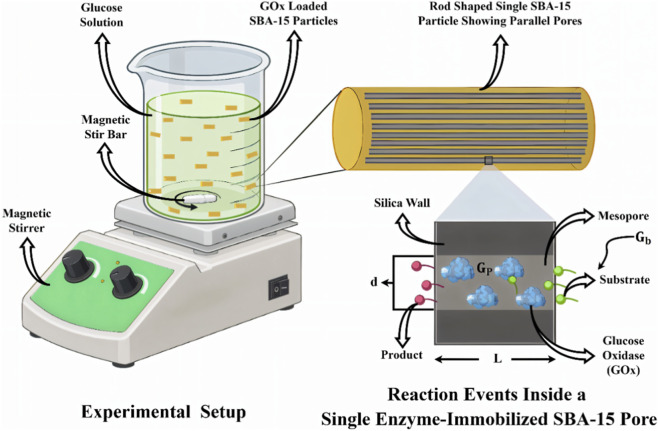
Schematic of the experimental configuration and the reaction-diffusion processes within a single GOx-immobilized SBA-15 particle: glucose transport from the bulk phase into the mesoporous structure and enzymatic conversion to products.

The mathematical modelling of the reaction-diffusion process for immobilized glucose oxidase (GOx) inside the mesoporous silica SBA-15 is considered for different loadings of the enzyme and different structures of the pores of the silica material. GOx enzyme is immobilized on the SBA-15 silica particles (for example, via electrostatic adsorption), acting as a biocatalyst for sensing glucose. Glucose oxidase (GOx) is a widely used enzyme for sensing glucose due to its high specificity towards glucose and high stability (Wilson and Turner, 1992). The enzyme catalyzes the oxidation of β-D-glucose in the presence of molecular oxygen (O_2_) to form D-glucono-δ-lactone and hydrogen peroxide (H_2_O_2_). This reaction occurs inside a batch reactor containing the silica particles with the immobilized enzyme that are well-mixed with the glucose solution. The process involves the diffusion of glucose into the silica particles, followed by its oxidation by the enzyme GOx. The reaction occurs inside the narrow cylindrical pores of SBA-15 silica material (with a diameter of approximately 6–12 nm), so internal diffusion of glucose into the enzyme may be the rate-limiting step. The kinetic mechanism of glucose oxidation by GOx is well-described by Michaelis–Menten kinetics, and the reaction scheme is illustrated conceptually (glucose + O_2_ → gluconic acid + H_2_O_2_) in the porous matrix. Figure 1 illustrates the experimental setup along with the reaction-diffusion process of glucose to gluconic acid and hydrogen peroxide inside a single SBA-15 particle with immobilized glucose oxidase (GOx).

The choice of rod and cuboid geometries in this study reflects the two morphologies most commonly reported for SBA-15 in the biosensing literature. Conventional hydrothermal synthesis of SBA-15 yields elongated rod-like particles with long, parallel cylindrical channels, whereas modified synthesis routes, including the platelet and short-channel variants reported by Chen et al. (2011), Ding et al. (2013), produce cuboid or platelet particles with substantially shorter diffusion paths. The two shapes represent the extremes of the parameters that can be considered for immobilised GOx applications: the rods will have a stronger diffusion limitation within the particles, but the cuboids will allow for the substrate to diffuse to the enzyme at a faster rate. Thus, being able to model and compare each of these shapes will allow for the contributions of each of these factors to the conversion of glucose to be determined.

To examine how intraparticle diffusion affects the reaction rate in a porous enzyme-carrier particle with uniformly distributed GOx, the geometry is modeled as one-dimensional along the pore length, and the following assumptions are adopted: (i) The transport of substrate and products through nanopores adheres to Fick’s law of diffusion, characterized by constant diffusivity and concentration gradients solely within the particle; (ii) the process is isothermal, with minimal heat effects and absent significant pressure gradients within the porous matrix; (iii) neither the substrate (glucose) nor the product (H_2_O_2_ or gluconic acid) impedes the enzyme’s activity, thus no substrate or product inhibition is incorporated into the kinetic model; (iv) electrostatic interactions between charged species and the silica support are negligible, indicating that reaction–diffusion is unaffected by internal electric fields; (v) the enzyme is uniformly distributed throughout the interior of the host particle, with GOx effectively immobilized within the mesoporous network; and (vi) glucose adsorption within the porous matrix is minimal relative to reaction and diffusion rates, rendering any transient accumulation of substrate inconsequential in the model formulation. These assumptions are generally made for enzyme immobilization systems and help to form the mass balance equations for the system.

The assumption of Fickian diffusion with a constant effective diffusivity warrants a brief justification given the confined environment inside SBA-15 pores. For the pore diameters considered in this study (6.8–11.4 nm) and a glucose molecule of hydrodynamic diameter of approximately 0.9 nm, the ratio of pore size to molecular size exceeds seven, which places the transport regime within hindered bulk diffusion rather than the Knudsen regime, since Knudsen behavior would require pore and molecular length scales to be comparable. Surface diffusion contributions are neglected on the basis of the low reported affinity of glucose for unmodified silica surfaces, consistent with the modelling assumptions adopted by Majumdar et al. (2016). The use of a single effective diffusivity also implicitly absorbs the effects of pore tortuosity and partial pore blocking by the immobilized enzyme into a lumped parameter, which is a standard practice for this class of immobilized-enzyme systems. For chemically modified SBA-15 analogues, however, such as the amino-functionalized supports of Khan et al. (2014) or the aluminosilicate variants of Fornerod et al. (2023), electrostatic interactions between glucose and the charged pore walls could introduce partitioning effects that are not captured by the present formulation, and a generalized Nernst-Planck treatment would be more appropriate in those cases. The Fickian model adopted here is therefore appropriate for unmodified SBA-15 under the conditions examined, but should be applied with caution to functionalized supports.

Beyond the diffusion assumptions discussed above, the present formulation also neglects electrostatic interactions between glucose and the silica framework, glucose adsorption on the pore walls, and any non-uniformity in the spatial distribution of immobilized GOx. Each of these simplifications reflects standard practice for unmodified SBA-15 in aqueous neutral-pH buffer. Glucose is a non-ionic sugar with no net charge under the operating conditions considered, so coupling between substrate transport and the local electric field at the silica surface is expected to be negligible, and electromigration terms can be omitted from the species balance. Glucose adsorption on bare silica is similarly weak relative to the diffusive and reactive fluxes encountered here, which justifies the absence of an explicit adsorption-desorption term in the governing equations and is consistent with the modeling choices adopted by Majumdar et al. (2016). The assumption of uniform GOx distribution corresponds to the typical outcome of solution-phase impregnation followed by drying, in which enzyme molecules access the mesoporous interior through diffusion-driven loading; spatial heterogeneity at the level of individual mesopores is not resolved by the present continuum description but is absorbed, together with tortuosity, into the lumped effective parameters. Within these limits, the model is most directly applicable to unmodified SBA-15 with covalently or physisorbed GOx, dilute glucose concentrations in aqueous neutral-pH buffer, and operating temperatures within the standard range for enzyme stability. Extensions to charged or strongly adsorbing substrates, ionic media, surface-functionalized silicas, or systems with deliberately engineered enzyme gradients would require additional terms in the governing equations and should be treated as outside the scope of the present formulation.

The governing equations are as follows (Majumdar et al., 2016):

In pore:

$$\frac{\partial G_{p}}{\partial t}=D\;\frac{\partial^{2}G_{p}}{{\partial x}^{2}}‐\;\frac{4}{d}\;V_{\max}\;\frac{G_{p}}{K_{m}\left(1+\frac{G_{b_{0}}‐\;G_{p}}{K_{i}}\right)+G_{p}}$$ (1)

In bulk:

$$\frac{dG_{b}}{dt}=\frac{N_{p}\;\pi \;d^{2}D}{2V}\;{\left(\frac{\partial G_{p}}{\partial x}\right)}_{x=0}$$ (2)

where $G_{p}$ denotes the concentration of glucose within the mesoporous structure; D is the glucose diffusion coefficient; and d indicates the pore diameter. The terms $V_{\max}$ and $K_{m}$ correspond to the maximum reaction rate and the Michaelis–Menten constant for free glucose oxidase, respectively. $K_{i}$ is the competitive inhibition constant; $G_{b_{0}}$ represents the initial concentration of glucose in the bulk solution; V denotes the total volume of the external liquid phase; $G_{b}$ is the glucose concentration in this surrounding bulk solution; and $N_{p}$ refers to the total number of pores that allow glucose to diffuse inward. Table 1 (Majumdar et al., 2016) summarizes the parameter values used in Equations 1, 2. The initial and boundary conditions corresponding to these equations are given in Equations 3–6:

**TABLE 1:** Physical and kinetic parameters used for the reaction–diffusion modeling of glucose oxidase immobilized in SBA-15 (Majumdar et al., 2016).

Parameters	Parameters Values		Units
	Rod SBA-15	Cuboid SBA-15	
d	6.8	11.4	$\left(\text{nm}\right)$
L	580	300	$\left(\text{nm}\right)$
$V_{\max}$	$8\times 10^{-7}$	$8\times 10^{-7}$	$\left(\text{mol}/s/\text{mg GOx}\right)$
$K_{m}$	1.23	1.23	$\left(\text{mM}\right)$
$G_{b_{0}}$	15	15	$\left(\text{mM}\right)$
$N_{p}$	$3.7\times 10^{16}$	$3.86\times 10^{16}$	$\left(\text{no}./g\right)$
V	20	20	$\left(\text{mL}\right)$
D	$2\times 10^{-14}$	$2\times 10^{-14}$	$\left(m^{2}/s\right)$
$K_{i}$ (at low Gox loading)	0.00492	0.01107	$\left(\text{mol}/m^{3}\right)$
$K_{i}$ (at high Gox loading)	0.01476	0.02091	$\left(\text{mol}/m^{3}\right)$

Initial at t=0:

$$G_{p}\left(x,0\right)=0\text{ for }0<x<L$$ (3)

$${\;G}_{b}\left(0\right)=G_{b_{0}\;}$$ (4)

Boundary conditions at x=0:

$$G_{p}\left(0,t\right)={\;G}_{b}\left(t\right)$$ (5)

at x=L:

$$G_{p}\left(L,t\right)={\;G}_{b}\left(t\right)$$ (6)

The equations can be expressed in a dimensionless form by introducing appropriate non-dimensional variables and parameters defined in Equation 7:

$$\left.\frac{\bar{G_{p}}=\frac{G_{p}}{G_{b_{0}}},\bar{x}=\frac{x}{L},\bar{G_{b}}=\frac{G_{b}}{G_{b_{0}}},\tau =\frac{D}{L^{2}}t,}{\Phi =\frac{4\;V_{\max}\;L^{2}}{d\;D\;K_{m}},\alpha =\frac{G_{b_{0}}}{\;K_{i}},\beta =\frac{G_{b_{0}}}{\;K_{m}},\gamma =\frac{N_{p}\;\pi \;d^{2}\;L}{\;2V},}\right\}$$ (7)

In this formulation, $\bar{G_{p}}$ and $\bar{G_{b}}$ are dimensionless concentrations corresponding to $G_{p}$ and $G_{b}$ , respectively. $\bar{x}$ is the dimensionless radial distance, τ is the dimensionless time, Φ is the Thiele modulus, α is the inhibition parameter, β is the saturation parameter, and γ denoted as the pore number-volume ratio. Table 2 (see Supplementary Appendix A), summarizes the dimensionless parameters for rod and cuboid SBA-15 geometries under varying GOx loadings.

**TABLE 2:** Dimensionless parameters for rod and cuboid SBA-15 geometries under varying GOx loadings.

Dimensionless parameters	Rod SBA-15		Cuboid SBA-15	
	low GOx loading	high GOx loading	low GOx loading	high GOx loading
Φ	$1.9306\times 10^{2}$	$7.7222\times 10^{2}$	$5.1348\times 10^{1}$	$1.3350\times 10^{2}$
α	$3.0488\times 10^{3}$	$1.0163\times 10^{3}$	$1.3350\times 10^{3}$	$7.1736\times 10^{2}$
β	12.1951	12.1951	12.1951	12.1951
γ	$1.1690\times 10^{-3}$	$1.1690\times 10^{-3}$	$1.772\times 10^{-3}$	$1.772\times 10^{-3}$

The parameter combinations summarized in Tables 1, 2 span two enzyme loading levels (low and high) for each particle geometry, corresponding to Thiele modulus values of Φ ≈ 51 for the weakest diffusion-limited case to Φ ≈ 772 for the strongest. This range was chosen to cover the regimes relevant for immobilized GOx biosensors reported in the literature (Fornerod et al., 2023; Khan et al., 2014; Majumdar et al., 2016): the lower Thiele modulus values correspond to surface-limited enzyme loading where substrate diffusion is fast relative to reaction, while the upper values represent high enzyme densities at which intraparticle diffusion becomes the dominant resistance. The initial bulk glucose concentration of 15 mM lies within the physiologically and analytically relevant window for glucose sensing, which typically extends from below 5 mM to above 20 mM depending on the application. The inhibition parameter α varies by approximately a factor of three between low and high loadings, consistent with the reported dependence of the effective inhibition constant on enzyme crowding (Shin et al., 2020).

The corresponding dimensionless forms of Equations 1, 2 are given below (see Supplementary Appendix B):

$$\frac{\partial \bar{G_{p}}}{\partial \tau }=\frac{\partial^{2}\bar{G_{p}}}{{\partial \bar{x}\;}^{2}}-\Phi \;\frac{\bar{G_{p}}}{1+\alpha \;\left(1-\bar{G_{p}}\right)+\beta \;\bar{G_{p}}}$$ (8)

In bulk:

$$\frac{d\bar{G_{b}}}{d\tau }=\gamma \;{\left(\frac{\partial \bar{G_{p}}}{\partial \bar{x}\;}\right)}_{\bar{x}=0}$$ (9)

The initial and boundary conditions corresponding to the dimensionless Equations 8, 9 are defined in Equations 10–13:

Initial conditions at τ=0:

$$\bar{G_{p}}\left(\;\bar{x},0\right)=0\text{ for }0<\bar{x}<1$$ (10)

$$\bar{G_{b}}\left(0\right)=1$$ (11)

Boundary conditions at $\bar{x}=0:$

$$\bar{G_{p}}\left(0,\tau \right)=\bar{G_{b}}\left(\tau \right)$$ (12)

at $\bar{x}=1:$

$$\bar{G_{p}}\left(1,\tau \right)=\bar{G_{b}}\left(\tau \right)$$ (13)

## 3 Materials and methods

Due to the complexity of the systems that must be solved analytically, there are few instances in which analytical solutions to reaction-diffusion systems exist. Additionally, while a variety of numerical approaches can be utilized to find the solutions to such systems, each requires a significant amount of computational power. Semi-analytical methods provide a benefit in that they reduce the dimensions of the problem while maintaining the principles of the systems. Semi-analytical methods reduce the complex governing equations to simpler algebraic or ordinary differential systems. This reduction yields efficient and accurate solutions at lower computational cost than fully numerical approaches.

Unlike purely numerical schemes, semi-analytical methods preserve the mathematical structure of the original problem. The resulting closed-form or near-closed-form expressions provide deeper physical insight and facilitate parametric analysis across a wide range of input conditions. These methods adapt well to linear and mildly nonlinear problems. They produce continuous and smooth solutions across the entire domain, free from the discretization errors and mesh-dependency issues associated with finite difference or finite element techniques. The semi-analytical Galerkin modal method is particularly effective in handling stiff, nonlinear systems characterized by spatial regularity and high convergence efficiency (Alfifi, 2021; Trefethen, 2000). The Galerkin formulation uses orthogonal sine basis functions to depict spatial dependence, resulting in a reduced-order system of ordinary differential equations that substantially decreases computational cost.

In Galerkin-based formulations, the initial step involves transforming the governing equations to ensure that the boundary conditions are homogeneous. The conversion is performed not due to the selection of a specific basis, but because Galerkin projection requires that the trial functions match the boundary conditions (Boyd, 2001; Trefethen, 2000). The initial pore concentration in Equation 8 satisfies the non-homogeneous Dirichlet boundary conditions specified in Equations 12, 13. The following transformation is applied:

$$v\left(\bar{x},\tau \right)=\bar{G_{p}}\left(\bar{x},\tau \right)-\bar{G_{b}}\left(\tau \right)$$ (14)

is applied to define a new function $v\left(\bar{x},\tau \right)$ that satisfies homogeneous boundary conditions $v\left(0,\tau \right)=v\left(1,\tau \right)=0$ . This step separates the boundary behavior from the rest of the PDE, enabling the problem to be expressed in terms of a set of basis functions that naturally vanish at the boundaries (Alfifi, 2021; Trefethen, 2000). Substituting the transformation from Equation 14 into the governing Equation 8 reformulates the system in terms of the new variable $v\left(\bar{x},\tau \right)$ , resulting in:

$$\frac{\partial v}{\partial \tau }=\frac{\partial^{2}v}{{\partial \bar{x}\;}^{2}}-S\left(\bar{G_{p}}\right)-\frac{\;d\bar{G_{b}}}{d\tau }$$ (15)

where $S\left(\bar{G_{p}}\right)=\Phi \;\frac{\bar{G_{p}}}{1+\alpha \;\left(1-\bar{G_{p}}\right)+\beta \;\bar{G_{p}}}\;.$ Upon establishing this homogeneous form, an approximate solution is formulated by representing it as a finite sum of basis functions:

$$v\left(\bar{x},\tau \right)\approx \sum_{j=1}^{N}a_{j}\left(\tau \right)v_{j}\left(\bar{x}\right),$$ (16)

where $a_{j}\left(\tau \right)$ represents the modal coefficients and $v_{j}\left(\bar{x}\right)$ denotes the selected trial functions. N represents the modal truncation order, indicating the number of sine modes preserved in the finite approximation. This study employs sine functions for $v_{j}\left(\bar{x}\right)=\sin\left(j\pi \bar{x}\right)$ , due to their orthogonality on the interval [0,1] and the boundary conditions $v_{j}\left(0\right)=v_{j}\left(1\right)=0$ , which render them suitable for spectral Galerkin projection (Boyd, 2001; Trefethen, 2000). The formulation effectively addresses the spatial domain analytically, while the temporal evolution is illustrated by the coefficients $a_{j}\left(\tau \right)$ leading to a reduced-order model that preserves the fundamental physics of the system (Alfifi, 2021). The original PDE system is reduced to a coupled set of ordinary differential equations (ODEs) by substituting this series into Equation 15 and applying the Galerkin condition. (Alfifi, 2021; Trefethen, 2000)

The semi-analytical Galerkin modal method shows many advantages compared to traditional methods. The modal method effectively captures global spatial behavior with a reduced number of degrees of freedom due to its spectral characteristics, unlike finite difference or finite element methods that need complete spatial discretization (Boyd, 2001; Trefethen, 2000). Moreover, methods like the Adomian Decomposition Method (ADM) and the Variational Iteration Method (VIM) can be complicated to apply due to their recursive nature. The Galerkin method, by contrast, allows direct integration of the nonlinear terms. This results in a method that is both flexible and stable to apply to the type of problem considered (Alfifi, 2021).

By applying the Galerkin condition to Equation 15, a system of modal equations is obtained, given in Equation 17 (see Supplementary Appendix C):

$$\frac{da_{m}}{d\tau }=‐{\left(\pi m\right)}^{2}a_{m}‐2F_{m}\left(\tau \right)‐2\gamma \sum_{j=1}^{N}\pi ja_{j}T_{m},\mathrm{for}\;\;m\;=1,2,\ldots ,N$$ (17)

where $T_{m}=\frac{1-{\left(-1\right)}^{m}}{m\pi }$ is the projection constant. The term $F_{m}\left(\tau \right)$ represents the nonlinear modal projection defined in Equation 18:

$$F_{m}\left(\tau \right)=\int_{0}^{1}S\left(\bar{G_{p}}\right)\;\sin\left(m\pi \bar{x}\right)d\bar{x},$$ (18)

this is evaluated numerically through high-resolution quadrature. The bulk concentration equation in Equation 9 is reformulated within the modal framework as given in Equation 19:

$$\frac{d\bar{G_{b}}}{d\tau }=\gamma \sum_{j=1}^{N}j\pi a_{j}$$ (19)

The initial deviation in pore concentration, defined as $v\left(\bar{x},0\right)=-1$ in the interior and zero at boundaries, is projected onto the modal basis, giving Equation 20:

$$a_{m}\left(0\right)=\left\{\begin{array}{cc} -\frac{4}{m\pi }, & \text{if }m\text{ is odd},\\ \;0, & \text{if }m\text{ is even}. \end{array}\right.$$ (20)

The bulk concentration is set as $\bar{G_{b}}\left(0\right)=1$ . The resulting system, consisting of N modal equations plus one bulk equation, is integrated over time using MATLAB’s stiff solver ode15s (see Supplementary Appendix D), with tight relative and absolute tolerances (MATLAB).

The Galerkin modal method presented here is efficient and accurate in solving the governing equations. Due to its computational performance and analytical balance, the method is ideal for use with nonlinear systems with dynamic boundaries (Alfifi, 2021; Boyd, 2001; Trefethen, 2000).

## 4 Results and discussion

The transport and reaction characteristics of glucose within SBA-15 carriers of both cuboid and rod morphologies were investigated under various enzyme loadings. Due to the low effective diffusivity of the porous substrate, steep concentration gradients develop within the substrate, especially at higher enzyme loadings. The Semi-analytical Galerkin modal method and the numerical Method of Lines (MOL) were employed to investigate the ability of the semi-analytical method to accurately reproduce the numerical results. A comparison of the computed solutions and the errors committed by the semi-analytical method and a sensitivity analysis of the parameters of the model are presented in the following subsections.

### 4.1 Behavior of glucose concentration in cuboid SBA-15

**FIGURE 2:**
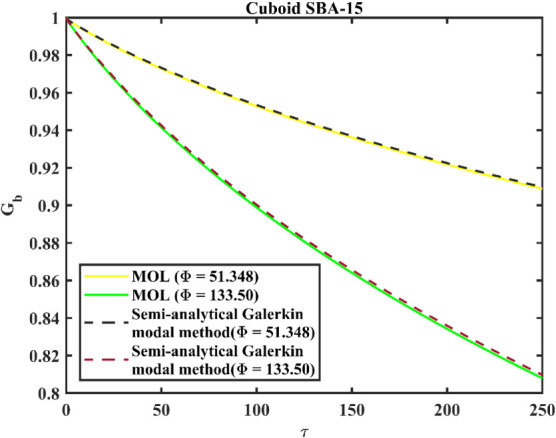
Bulk glucose concentration profiles in a cuboid SBA-15 domain at low (Φ = 51.3) and high (Φ = 133.5) GOx loadings, computed by the Method of Lines (MOL) and the semi-analytical Galerkin modal method.

Within the cuboid geometry, the glucose concentration profiles show progressive depletion of glucose throughout the pore space, with the extent of depletion increasing as the enzyme loading increases. At low loadings of the enzymes, the decrease in glucose within the pores is relatively smooth. However, at higher loadings, the rate of reaction is more dominant than the diffusion of the glucose molecules, leading to a greater depletion of glucose within the inner regions of the pore. These trends are exhibited in Figure 2, which depicts the concentration curves for both the semi-analytical Galerkin modal method and the MOL method for the cuboid geometry. The two curves are nearly indistinguishable from one another, indicating that the truncated series of eigenmodes is able to accurately describe the reaction within the cuboid geometry.

Further confidence in this conclusion can be drawn from the values of the differences between these two methods, reported in Tables 3, 4. The absolute differences in concentrations between these two methods are, again, extremely small, varying only between about 10^−4^ and 10^−3^ in most of the region. Even at the regions where the concentration declines more rapidly with increasing enzyme loading, the relative error is only a few percent. The error is slightly higher at the center of the pore, due to the very low concentrations in that region of the pore. Thus, the similar concentration profiles and low errors observed in both methods indicate that the cuboid geometry does not hinder convergence of the semi-analytical method.

**TABLE 3:** Comparative error analysis for low-loading cuboid SBA-15 case ( Φ = 51.348).

τ	Numerical (MOL)	Semi-analytical	Absolute error	Relative error %
0	1	1	0	0
62.5	0.9674	0.96792	0.00052621	0.054394
125	0.9442	0.94488	0.0006824	0.072273
187.5	0.92521	0.92598	0.00076862	0.083075
250	0.90888	0.90971	0.00082787	0.091087

**TABLE 4:** Comparative error analysis for high-loading cuboid SBA-15 case ( Φ = 133.50).

τ	Numerical (MOL)	Semi-analytical	Absolute error	Relative error %
0	1	1	0	0
62.5	0.92986	0.93107	0.0012105	0.13018
125	0.88076	0.88228	0.0015189	0.17245
187.5	0.84134	0.84301	0.0016702	0.19852
250	0.80799	0.80975	0.0017636	0.21827

### 4.2 Behavior of glucose concentration in rod SBA-15

**FIGURE 3:**
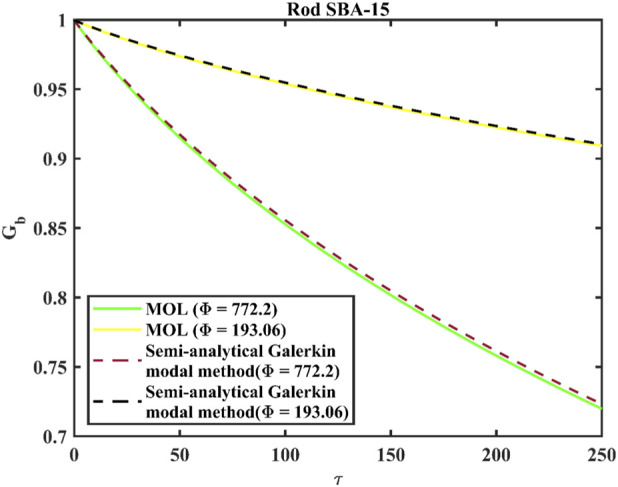
Bulk glucose concentration profiles in a rod SBA-15 domain at low (Φ = 193.06) and high (Φ = 772.2) GOx loadings, computed by the Method of Lines (MOL) and the semi-analytical Galerkin modal method.

The rod-shaped SBA-15 particles exhibit a stronger diffusion limitation than the cuboid particles due to their narrower pore diameter and longer channel length. Consequently, the glucose concentration decreases much more steeply within the SBA-15 particles, particularly at high enzyme loadings (Figure 3). Despite these diffusion limitations, the semi-analytical Galerkin and MOL curves remain closely matched, confirming that the method retains accuracy even in this regime.

The comparison of the quantitative errors of these two methods, presented in Tables 5, 6, demonstrates that though the discrepancies between the two methods are slightly higher than in the case of the cuboid system, the errors remain small. As before, the largest errors occur in regions with high concentration gradients and low substrate concentrations. However, these discrepancies remain within acceptable limits, and there are no trends towards either under- or over-prediction of the behavior of the particles in these systems. Thus, both methods accurately reflect the strong confinement effect exhibited by the rod SBA-15 system.

**TABLE 5:** Comparative error analysis for low-loading rod SBA-15 case ( Φ = 193.06).

τ	Numerical (MOL)	Semi-analytical	Absolute error	Relative error %
0	1	1	0	0
62.5	0.96835	0.96907	0.00071206	0.073533
125	0.9452	0.9461	0.00090542	0.095791
187.5	0.92596	0.92696	0.001004	0.10843
250	0.90925	0.91032	0.001067	0.11735

**TABLE 6:** Comparative error analysis for high-loading rod SBA-15 case ( Φ = 772.2).

τ	Numerical (MOL)	Semi-analytical	Absolute error	Relative error %
0	1	1	0	0
62.5	0.89809	0.90062	0.0025242	0.28106
125	0.82615	0.82922	0.0030787	0.37265
187.5	0.7684	0.77169	0.0032939	0.42867
250	0.71976	0.72315	0.0033876	0.47066

### 4.3 Comparative error evaluation

Tables 3–6 show that the semi-analytical and numerical methods are consistent across all morphologies and loadings. The largest deviations between the two methods exist at longer times and higher loadings, but are still very small. Furthermore, the percentage of relative error between the methods ranges from 0% to about 0.47%, indicating that the error is quite low and does not meaningfully impact the results of the analysis. The expected sensitivity to changes in substrate concentration is reflected in the rise of the deviation within the internal pore region. Furthermore, no method exhibits a bias relative to the MOL results, with the Galerkin method providing good agreement with the MOL solution within two of the pore geometries. Thus, the semi-analytical method provides a computationally efficient method of calculating the transport that is as accurate as the numerical approach.

It is worth noting that the favorable agreement observed under diffusion-limited conditions is in part due to the expected behavior of the modal formulation alone; it is physically intuited that under these conditions, the reaction term is weak relative to the diffusion term, and thus its contribution to the spatial structure is negligible. Under kinetically-limited regimes, however, the reaction term becomes “stiffer than the diffusion term, and the gradients associated with the reaction are restricted to thin boundary layers within the rods. Consequently, a greater number of modes will be required to adequately represent these spatial gradients. As stated above, though, computations using only N = 20 modes was sufficient to represent the Method of Lines solutions to within sub-percent error for each of the four sets of parameters, even in the diffusion-limited case with the highest loading of rods ( Φ = 772). For problems with substantially sharper internal gradients - such as those involving fast irreversible reactions or considerably higher Thiele modulus values than those considered here - adaptive mode selection or a hybrid scheme combining modal expansion with local refinement near the boundaries would be more appropriate than uniform modal truncation.

### 4.4 Validation against experimental data

To assess the predictive capability of the present framework beyond method-to-method verification, the model was compared against experimental bulk glucose depletion data reported by Majumdar et al. (2016) for the low-loading rod SBA-15 case (Φ = 193.06). The experimental points were extracted from the published figure using WebPlotDigitizer, and the experimental times were converted to the dimensionless time $\tau =\frac{D}{L^{2}}t$ using the parameters of Table 1. Five representative points spanning the early-to-intermediate portion of the simulated window were selected, and both the Method of Lines and the semi-analytical Galerkin solutions were evaluated at the matched τ values. The comparison is summarized in Table 7.

**TABLE 7:** Validation of the reaction–diffusion model against experimental data from Majumdar et al. (2016) for low-loading rod SBA-15 (Φ = 193.06).

τ	Experimental (Majumdar et al., 2016)	Numerical (MOL)	Semi-analytical	Absolute error (MOL)	Relative error % (MOL)	Absolute error (Semi-analytical)	Relative error % (Semi-analytical)
0	0.9950	1	1	0.0050	0.503	0.0050	0.503
41.84	0.9616	0.9768	0.9780	0.0152	1.576	0.0164	1.700
86.08	0.9495	0.9582	0.9598	0.0087	0.919	0.0103	1.087
127.95	0.9351	0.9433	0.9451	0.0082	0.881	0.0100	1.072
213.28	0.9156	0.9178	0.9198	0.0022	0.243	0.0042	0.464

The agreement between model and experiment is close across the sampled window. The mean absolute percentage error is 0.82% for the Method of Lines and 0.97% for the semi-analytical Galerkin method, with root-mean-square deviations of 0.009 and 0.010 respectively. These values lie within the combined uncertainty of graphical digitization (approximately ±2% based on pixel resolution) and the published uncertainty in the kinetic and transport parameters.

Two points follow from this comparison. First, the near-identical accuracy of the two methods against the experimental curve confirms that the reduced-order Galerkin formulation does not lose predictive fidelity relative to MOL, reinforcing the internal method-comparison of Sections 4.1 and 4.2. Second, the quantitative match with the experimental depletion profile supports the applicability of the Michaelis-Menten reaction-diffusion framework with competitive product inhibition, as parameterized in (Majumdar et al., 2016), for describing transport-limited glucose conversion in immobilized GOx systems under moderate enzyme loading.

The largest single-point deviation of approximately 1.7% occurs at τ = 41.84, within the initial transient where the internal field is still adjusting from the zero-interior condition. Agreement improves once the system relaxes into its slowly-evolving regime, with relative errors dropping below 0.5% at later sampled points. Extension of the validation to crowded high-loading conditions, where reported kinetic parameters carry additional uncertainty due to enzyme crowding effects (Shin et al., 2020), is identified as future work. The present comparison nevertheless establishes that, within the regime of primary practical interest for glucose sensing applications, the model yields quantitative predictions consistent with independent experimental observations. Accordingly, claims of broader applicability across the full SBA-15 parameter space, including the rod high-loading, cuboid low-loading, and cuboid high-loading regimes, should be interpreted within the scope of this single-case validation; full multi-regime validation, requiring controlled experimental measurements at well-characterized enzyme loadings and particle morphologies, is left for future work.

### 4.5 Sensitivity analysis

The influence of each of the transport-reaction parameters upon the conversion of glucose can be assessed through the determination of the normalized local sensitivity of the system’s performance to each of those parameters. Each of these analyses provides insight into which of the physical or kinetic processes within the system are most influential under the conditions of the cuboid SBA-15 structure. The analysis of each of these two parameters reveals a difference in their influence upon the system’s performance.

**FIGURE 4:**
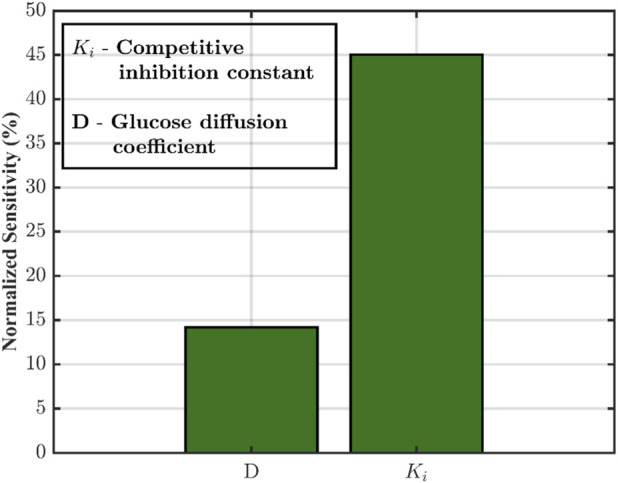
Normalized sensitivity of glucose conversion (%) to the effective diffusivity and competitive inhibition constant for the cuboid SBA-15 system.

As shown in Figure 4 ±10% variation in the inhibition constant leads to a significantly major change in conversion than an equivalent relative variation in diffusivity. The response to changes in $K_{i}$ is significant, with conversion shifting by nearly 45%, while shifts in D lead to a more modest variation of about 14%. Both sensitivities show positive signs, implying that increases in either diffusivity or the inhibition constant augment overall glucose consumption. This behavior is consistent with the underlying physics: accelerated substrate migration through the mesoporous channels is promoted by an increase in D , whereas the competitive inhibition strength linked to the accumulation of reaction products is reduced by a higher value.

In spite of these advantageous contributions, the extent of the normalized sensitivity related to $K_{i}$ is evidently predominant. This finding shows that, within the diffusivity regime relevant to the cuboid SBA-15 particles, the system is nearing the transition from diffusion-controlled to reaction-influenced behavior. Consequently, small changes in diffusivity lead to small changes in conversion. However, changes in the inhibition constant directly influence the degree of inhibition of the enzyme along the pore walls, indicating that it is an influential control variable for the system. Thus, the influence of the inhibition constant indicates the importance of inhibitory kinetics for the system, while the relatively lesser influence of the diffusivity indicates the secondary importance of diffusive transport to the system. Physically, the dominance of $K_{i}$ in this regime reflects that overall glucose conversion is rate-limited by enzyme turnover rather than substrate transport once intraparticle gradients are mild. At the Thiele modulus values examined here, the local glucose concentration along the pore is sufficient to keep most enzyme active sites near saturation, so further increases in effective diffusivity bring only marginal additional substrate to already-occupied sites. The inhibition constant, by contrast, directly controls the fraction of those active sites that remain catalytically productive at the prevailing local product concentration, and small variations in $K_{i}$ therefore translate into proportionally larger variations in the rate of net glucose consumption. This regime-dependent behavior also implies that the relative dominance of $K_{i}$ over the effective diffusivity would diminish for systems operating at substantially lower Thiele modulus, where transport begins to limit substrate availability at the active sites; the sensitivity result reported here is therefore specific to the diffusion-limited regime characteristic of typical immobilized-GOx biosensors.

Overall, the fact that $K_{i}$ is dominant over D has some consequences for biosensor design. The inhibition constant indicates the degree to which the accumulated gluconolactone molecules inhibit the enzymes along the walls of the pores. The effect of this inhibition upon the biosensor response is twofold. For instance, one effect of inhibition is that it limits the range over which the biosensor exhibits a linear response to the concentration of the analyzed substance; at high concentrations of the substance to be analyzed, the biosensor saturates and can no longer exhibit increases in the measured current. Second, it affects the transient response time, since the build-up of inhibiting product inside the pore is a slow intraparticle process relative to pure diffusion-reaction equilibration, so a small reduction in $K_{i}$ can appreciably lengthen the time required to reach a stable output signal. The present sensitivity result therefore suggests that, within the diffusivity regime relevant to the cuboid SBA-15 particles examined here, efforts to improve biosensor performance through nanostructure engineering of the diffusion path - for instance by reducing channel length or widening pores - will yield diminishing returns unless accompanied by strategies that mitigate product inhibition. Such strategies include co-immobilization with catalase or HRP (Dai et al., 2008; Pitzalis et al., 2017) to remove H_2_O_2_ and limit gluconolactone accumulation, or the use of enzyme variants engineered for reduced sensitivity to inhibitory products.

### 4.6 Implications for biosensor design

The results of the preceding subsections can be translated into concrete design guidance for mesoporous glucose biosensors in three respects. First, the predicted bulk depletion curves allow enzyme loading and particle geometry to be matched to a target substrate concentration range and response time, rather than established by trial-and-error experimentation: the simulations show that rod particles at high GOx loading achieve deeper conversion within the same residence time but at the cost of steeper internal gradients, which can broaden the effective response window while introducing diffusion-limited delays near the pore center. Second, the normalized sensitivity results indicate that, within the diffusivity regime examined here, efforts directed toward mitigating product inhibition - for example, through co-immobilization with catalase or HRP (Dai et al., 2008; Pitzalis et al., 2017) or through the use of enzyme variants with reduced $K_{i}$ sensitivity - are expected to produce larger performance gains than further optimization of pore geometry alone, providing concrete prioritization guidance for experimental development. Third, the reduced-order structure of the Galerkin modal formulation is itself useful as a forward model for inverse-design problems, since only a small number of retained modal coefficients are required to reproduce the full concentration field, enabling the formulation to be embedded in optimization loops targeting specific device criteria such as linear response range or limit of detection - a computational task that would be impractical with a full MOL solver. Together, these connections situate the present framework as a quantitative working tool for guiding mesoporous biosensor development.

## 5 Conclusion

This study applies the semi-analytical Galerkin modal method to nonlinear reaction-diffusion problems in glucose oxidase-immobilized SBA-15 materials. The method exhibits a strong correlation to the solutions obtained from the method of lines for several different pore geometries and enzyme loadings. The Galerkin modal method provides an accurate representation of the reaction-diffusion processes with significantly reduced computational complexity relative to the numerical methods. Beyond the internal method-to-method comparison, the model reproduces independently reported experimental depletion data (Majumdar et al., 2016) with mean absolute percentage error below 1%, providing external validation of the reaction–diffusion framework under the operating conditions examined. The model investigates the significant impact of several different factors on the glucose transport and conversion in the immobilized enzyme systems. The influence of pore geometry, enzyme loading, and inhibitory effects on glucose transport and conversion is examined. The results of the study indicate that competitive inhibition has a significantly greater influence on the conversion of glucose than the effective diffusivity of the pores of the mesoporous material. The Galerkin modal approach is proven to be an effective means of performing a systematic analysis of glucose oxidase-immobilized materials. The present model predicts glucose conversion in these systems and provides design insights for such catalysts and sensors. Specifically, the model outputs inform biosensor design choices regarding particle geometry selection, enzyme loading, and the prioritization of inhibition-mitigation strategies.

## Data Availability

The original contributions presented in the study are included in the article/Supplementary Material, further inquiries can be directed to the corresponding author.

## Nomenclature

$G_{p}$: Glucose concentration inside the mesoporous particle $\left(\text{mol}/m^{3}\right)$
$G_{b}$: Glucose concentration in the bulk solution $\left(\text{mol}/m^{3}\right)$
$G_{b_{0}}$: Initial bulk glucose concentration $\left(\text{mol}/m^{3}\right)$
D: Effective diffusion coefficient of glucose in the pore $\left(m^{2}/s\right)$
$N_{p}$: Number of pores available for diffusion $\left(\text{no}./g\right)$
V: Volume of the bulk liquid phase $\left(\text{mL}\right)$
$K_{i}$: Competitive inhibition constant $\left(\text{mol}/m^{3}\right)$
d: Pore diameter of SBA-15 $\left(\text{nm}\right)$
L: Effective pore length $\left(\text{nm}\right)$
$V_{\max}$: Maximum reaction rate of glucose oxidase $\left(\text{mol}/s/\text{mg GOx}\right)$
$K_{m}$: Michaelis–Menten constant $\left(\text{mM}\right)$
x: Spatial coordinate along the pore length(m)
t: Time(s)
$a_{j}\left(\tau \right)$: Galerkin modal coefficient
$v\left(\bar{x},\tau \right)$: Deviation of pore glucose concentration

### Dimensionless parameters (Greek symbols)

$\bar{G_{b}}=\frac{G_{b}}{G_{b_{0}}}$: Dimensionless bulk concentration
$\bar{G_{p}}=\frac{G_{p}}{G_{b_{0}}}$: Dimensionless pore concentration
$\bar{x}=\frac{x}{L}$: Dimensionless spatial coordinate
$\tau =\frac{D}{L^{2}}t$: Dimensionless time
$\Phi =\frac{4\;V_{\max}\;L^{2}}{d\;D\;K_{m}}$: Thiele modulus
$\alpha =\frac{G_{b_{0}}}{\;K_{i}}$: Inhibition parameter
$\beta =\frac{G_{b_{0}}}{\;K_{m}}$: Saturation parameter
$\gamma =\frac{N_{p}\;\pi \;d^{2}\;L}{\;2V}$: Pore number–volume ratio

### Abbreviations

GOx: Glucose oxidase
SBA-15: Santa Barbara Amorphous-15 mesoporous silica
PDE: Partial differential equation
ODE: Ordinary differential equation
MOL: Method of Lines
ADM: Adomian Decomposition Method
VIM: Variational Iteration Method
HRP: Horseradish peroxidase
MCM-41: Mobil Composition of Matter-41
MCF: Mesocellular foam
