# Supplementary material for: Semi-analytical Galerkin modeling of glucose transport and reaction in glucose oxidase-immobilized SBA-15 mesoporous silica
Source: Front Chem. 2026 Jun 9;14:1848563. doi: 10.3389/fchem.2026.1848563 (PMC13287025; doi:10.3389/fchem.2026.1848563)
Supplement: Supplementary file 1 [file DataSheet1.doc]

# Appendix

**Appendix A**

**Framework for deriving dimensionless parameters presented in Table 2.**

All variables presented in Table 1 were converted to a consistent set of SI units to eliminate discrepancies from mixed unit systems and to establish a uniform basis for calculating the dimensionless parameters in Table 2.

**Rod SBA-15**

1mL =
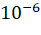

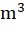
:

*V* = 20 mL = 20
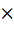

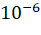

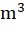
 = 2
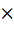

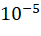

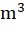
.

1
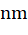
 =
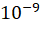
m:


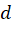
 = 6.8 nm = 6.8
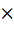

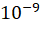
m and L = 580 nm = 580
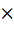

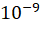
m = 5.8
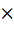

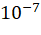
m.

1mM = 1
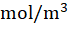
:


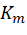
= 1.23 mM = 1.23
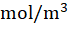
 and


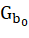
= 15 Mm = 15
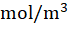
.

Mass = 0.015 g:


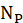
 = 3.7
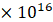

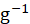

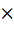
 0.015 g = 5.55
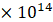
 pores.

**Low GOx loading case:**

Convert the Table 1
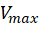
 per mg GOx to a per area value by multiplying by GOx surface loading (for low GOx loading take 0.03 mg
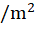
):


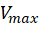
 = 8
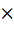

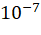
 mol
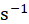
m
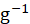

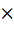
 0.03 mg
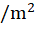
 = 2.4
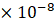
 mol
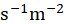


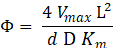


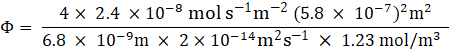


=
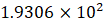


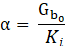


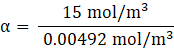


**=** 3.0488
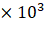


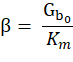


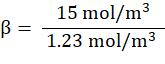


= 12.1951


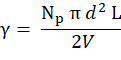


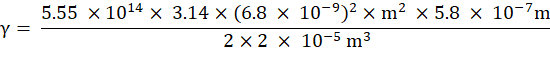


= 1.1690
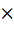

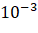
.

**High GOx loading case:**

Convert the Table 1
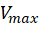
 per mg GOx to a per area value by multiplying by GOx surface loading (for high GOx loading take 0.12 mg
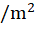
):


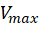
 = 8
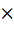

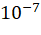
 mol
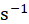
m
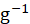

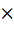
 0.12 mg
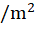
 = 9.6
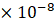
 mol
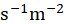


The parameters β and γ were adopted directly from the low-rod configuration, as their definitions and numerical values remain unchanged for the present analysis.


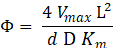


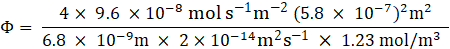


= 7.7222
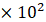


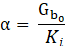


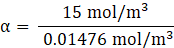


**=** 1.0163
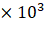
.

**Cuboid SBA-15**

For the cuboid configuration, the parameters
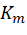
,
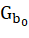
,*V*, and
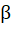
 were taken to be the same as those used in the rod case, as their definitions and required input values remain unchanged.

1
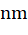
 =
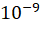
m:


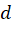
 = 11.4 nm = 1.14
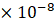
 m and L = 300 nm = 300
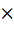

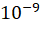
m = 3
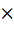

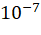
m.

Mass = 0.015 g:


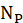
 = 3.86
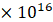

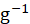

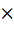
 0.015 g = 5.79
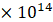
 pores.

**Low GOx loading case:**

Convert the Table 1
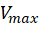
 per mg GOx to a per area value by multiplying by GOx surface loading (for low GOx loading take 0.05 mg
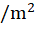
):


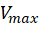
 = 8
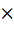

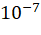
 mol
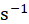
m
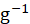

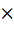
 0.05 mg
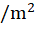
 = 4
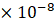
 mol
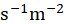


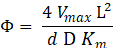


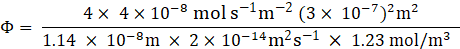


= 5.1348
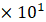


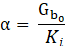


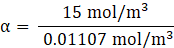


**=** 1.3350
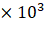


= 1.772 .

**High GOx loading case:**

Convert the Table 1 per mg GOx to a per area value by multiplying by GOx surface loading (for high GOx loading take 0.13 mg):

= 8 mol m 0.13 mg = 1.04 mol

The parameter γ were adopted directly from the cuboid low-rod configuration, as their definitions and numerical values remain unchanged for the present analysis.

= 1.3350

**=** 7.1736 .

**Appendix B**

**Derivation of the dimensionless forms of equations (1) and (2)**

Let

Let = then equations (1):

Substituting [B.2] and [B.3] in above equation we have:

Now take

Let

Now substitute this in [B.5] we get equation (8):

Substituting [B.1] and [B.4] in equation (2) we have:

Let we get equation (9):

The initial and boundary conditions are nondimensionalized following the same approach.

**Appendix C**

**Semi-analytical Galerkin modal method.**

The semi-analytical Galerkin modal method represents a reduced-order technique that converts a spatially distributed diffusion-reaction problem into a finite system of ordinary differential equations via modal expansion. The method is classified as semi-analytical due to the analytical treatment of spatial dependence in pore concentration through predefined basis functions, whereas the resulting time-dependent system is addressed numerically. This formulation decomposes the pore concentration into a bulk component and a spatial deviation, resulting in homogeneous boundary conditions for the deviation field. Given these boundary conditions, a selection of trial functions is made to ensure that the deviation inherently meets the specified constraints. The deviation is subsequently expressed as a truncated series of sine functions, which vanish at the boundaries and serve as eigenfunctions of the diffusion operator. This selection precisely enforces the boundary conditions and diagonalizes the diffusion term, facilitating an analytical reduction of the governing equations to a system of modal ordinary differential equations. Nonlinear reaction terms are integrated via explicit projections onto the modal basis, followed by temporal integration of the reduced system using a numerical ODE solver.

The sine basis was chosen following a comparative assessment, as it demonstrated stable convergence and a high degree of agreement with the numerical solution in contrast to alternative bases. Other trial functions, including polynomial functions, can be employed within the Galerkin framework; however, they did not produce results that closely aligned with the numerical solution for similar truncation orders. The sine expansion provided improved accuracy and convergence with a reduced number of modes and was utilized in this study.

Let in equation (8) we have:

Set

which gives homogeneous boundary conditions (since and ). Substitute [C.2] in [C.1] we have:

Choose a trail function , where = 1, 2, …, . They are orthogonal and satisfies . The solution is approximated by truncating the expansion to a finite number of modes as follows:

and

.

Define residual as:

Using Galerkin condition:

Substituting [C.3] in [C.4] we have:

By orthogonality property of sine function we have:

Similarly,

We know that

since

Define:

Substituting [C.6], [C.7], [C.8], and [C.9] in [C.5] we have:

Now from we have

Substituting [C.11] in equation (9) the bulk ODE becomes:

Substituting [C.12] in [C.10] we have:

We get ODEs for and , now to integrate these ODEs we need initial conditions for all unknowns that is and . We know that , to find we use [C. 2] at .

We have multiple both sides by and integrate over [0,1] we have:

Thus

The resulting system, consisting of modal equations in [C.13] plus one bulk equation in [C.12], is integrated over time using MATLAB’s stiff solver ode15s.

**Appendix D**

**Matlab program to solve modal equations in [C.13] plus one bulk equation in [C.12].**

Here giving code for the cuboid SBA-15 case, by changing values of alpha, beta, gamma, and phi using Table 2 we get rod SBA-15 case.

alpha_list = [1.3350*10^3, 7.1726*10^2];

beta_list = [12.1951, 12.1951];

gamma_list = [1.772*10^-3, 1.772*10^-3];

phi_list = [5.1348*10 , 1.3350*10^2];

N = 20;

nx = 2000;

xq = linspace(0,1,nx)';

wmat = sin((1:N)' * (pi*xq'));

S = (1 - (-1).^(1:N))./((1:N)*pi);

theta = 1.00;

tspan = [0 250];

if isempty(get(groot,'CurrentFigure'))

figure; hold on;

else

hold on;

end

colors = cool(numel(alpha_list));

for k = 1:numel(alpha_list)

alpha = alpha_list(k);

beta = beta_list(k);

gamma = gamma_list(k);

phi = phi_list(k);

a0 = zeros(N,1);

for n=1:N

if mod(n,2)==1

a0(n) = -4/(n*pi);

end

end

Cb0 = 1;

y0 = [a0; Cb0];

opts = odeset('RelTol',1e-10,'AbsTol',1e-12);

[tau,y] = ode15s(@(t,y) rhs_modal_corrected(t,y,phi,alpha,beta,gamma,N,xq,wmat,S,theta), ...

tspan,y0,opts);

Cb = y(:,end);

plot(tau,Cb,'LineWidth',1.6,'Color',colors(k,:), ...

'DisplayName', sprintf('semi-analytical Galerkin modal method (\\phi = %.3g))', phi));

end

xlabel('\tau'); ylabel('G_b');

legend('Location','best');

box on;

title('cuboid SBA-15');

function dy = rhs_modal_corrected(~,y,phi,alpha,beta,gamma,N,xq,wmat,S,theta)

a = y(1:N);

Cb = y(N+1);

w_at_x = (a.' * wmat).'; % (nx x 1) column vector

Cp_at_x = w_at_x + Cb;

den = 1 + alpha*(1 - Cp_at_x) + beta*Cp_at_x;

R = phi .* Cp_at_x ./ den;

Fm = zeros(N,1);

for m=1:N

phi_m = wmat(m,:)';

Fm(m) = trapz(xq, R .* phi_m);

end

Fm = theta * Fm;

lap = - (pi*(1:N)').^2 .* a;

sum_n = sum((1:N)'.*pi .* a);

da = lap - 2*Fm(:) - 2*gamma*sum_n.*S(:);

dCb = gamma * sum_n;

dy = [da; dCb];

end

**Appendix E**

**Matlab program to obtain the numerical solution** (**Method of Lines (MOL)**) **for the equations (8) and (9) satisfying the initial and boundary conditions in equations (10) – (13).**

Here giving code for the cuboid SBA-15 case, by changing values of alpha, beta, gamma, and phi using Table 2 we get rod SBA-15 case.

Nx = 81;

L = 1.0;

x = linspace(0, L, Nx)';

dx = x(2) - x(1);

nInt = Nx - 2;

tau_max = 250;

tspan = [0, tau_max];

opts = odeset('RelTol',1e-6,'AbsTol',1e-8,'MaxStep',1.0);

alpha_list = [1.3350*10^3, 7.1726*10^2];

beta_list = [12.1951, 12.1951];

gamma_list = [1.772*10^-3, 1.772*10^-3];

phi_list = [5.1348*10 , 1.3350*10^2];

if isempty(get(groot,'CurrentFigure'))

figure; hold on;

else

hold on;

end

colors = prism(numel(alpha_list));

linestyles = {'--','--'};

for cs = 1:numel(alpha_list)

alpha = alpha_list(cs);

beta = beta_list(cs);

gamma = gamma_list(cs);

phi = phi_list(cs);

Cb0 = 1.0;

Cp0_full = zeros(Nx,1);

Cp0_full(1) = Cb0;

Cp0_full(end) = Cb0;

Cp0_full(2:end-1) = 0;

y0 = [ Cp0_full(2:end-1) ; Cb0 ];

params.alpha = alpha;

params.beta = beta;

params.gamma = gamma;

params.phi = phi;

params.dx = dx;

params.nx = Nx;

params.nint = nInt;

sol = ode15s(@(t,y) mol_rhs(t,y,params), tspan, y0, opts);

tplot = linspace(0, tau_max, 400);

Y = deval(sol, tplot);

Cb_sol = Y(end,:);

plot(tplot, Cb_sol, 'Color', colors(cs,:), 'LineStyle', linestyles{cs}, ...

'LineWidth',1.6, ...

'DisplayName', sprintf('MOL (\\phi = %.2f)', phi));

end

xlabel('\tau (dimensionless time)');

ylabel('G_b (dimensionless)');

legend('Location','best');

box on;

xlim([0 tau_max]);

function dydt = mol_rhs(~, y, p)

nInt = p.nint;

dx = p.dx;

Cp_int = y(1:nInt);

Cb = y(end);

Cp = zeros(p.nx,1);

Cp(1) = Cb;

Cp(2:end-1) = Cp_int;

Cp(end) = Cb;

Cp_left = Cp(1:end-2);

Cp_mid = Cp(2:end-1);

Cp_right = Cp(3:end);

d2Cp_dx2 = (Cp_left - 2*Cp_mid + Cp_right) / dx^2;

denom = 1 + p.alpha*(1 - Cp_mid) + p.beta*Cp_mid;

denom(denom <= 1e-14) = 1e-14;

R = p.phi * Cp_mid ./ denom;

dCpdt_int = d2Cp_dx2 - R;

dCpdx_at0 = (Cp(2) - Cp(1)) / dx;

dCbdt = p.gamma * dCpdx_at0;

dydt = [ dCpdt_int; dCbdt ];

end
